# Glucocorticoid receptor positively regulates transcription of *FNDC5* in the liver

**DOI:** 10.1038/srep43296

**Published:** 2017-02-27

**Authors:** Hyoung Kyu Kim, Yu Jeong Jeong, In-Sung Song, Yeon Hee Noh, Kyo Won Seo, Min Kim, Jin Han

**Affiliations:** 1 National Research Laboratory for Mitochondrial Signaling, Department of Physiology, Department of Health Sciences and Technology, BK21 plus Project Team, College of Medicine, Cardiovascular and Metabolic Disease Center, Inje University, Busan, Korea; 2 Department of Integrated Biomedical Science, College of Medicine, Inje University, Busan, Korea; 3 Department of Biomedical Sciences, College of Medicine, Ulsan University, Asan Medical Center, Republic of Korea

## Abstract

Irisin is secreted by skeletal muscle during exercise and influences energy and metabolic homeostasis. This hormone is a cleaved and secreted fragment of fibronectin type III domain-containing 5 (FNDC5). Elucidation of the *FNDC5* gene regulation mechanism is necessary to clarify the function of irisin as a potential therapeutic target in human metabolic diseases. Thus, we investigated the genetic and epigenetic mechanisms that regulate expression of the *FNDC5* gene. *FNDC5* mRNA was strong expressed in major energy-dependent human tissues, including heart, brain, liver, and skeletal muscle. Promoter analysis of the *FNDC5* gene revealed that the core promoter region of the *FNDC5* gene contained one CpG island that was located just upstream of the transcriptional start site for variants 2 and 3. Treatment with the histone deacetylase inhibitor sodium butyrate and the demethylating agent 5-azacytidine increased mRNA expression of *FNDC5* in Huh7 cells. Prediction of transcription factor binding sites suggested that the glucocorticoid receptor was involved in the regulation of *FNDC5* expression, and indeed, cortisol treatment increased mRNA expression of *FNDC5* in Huh7 cells. Collectively, these findings offer insight into the genetic and epigenetic regulation of *FNDC5*, providing the initial steps required for understanding the role of irisin in the metabolic homeostasis.

Recently, a new muscle-derived hormone, irisin, was reported. This protein is secreted from skeletal muscle and induced by overexpression of peroxisome proliferator-activated receptor gamma coactivator 1-alpha (PGC-1α)^1^. Irisin is produced as a result of cleavage of the membrane protein fibronectin type III domain-containing protein 5 (FNDC5) and contributes to maintenance of metabolic homeostasis via induction of the “browning” of white adipocytes through increased expression of uncoupling protein 1 (UCP1), leading to increased energy expenditure^1^,^2^,^3^. Over the past 2 years, multiple studies on *FNDC5* and the novel hormone in both rodents and humans have been undertaken^3^,^4^,^5^,^6^,^7^. Recently, the diverse role of *FNDC5*/irisin in diseases, such as diabetes^8^,^9^, hypothyroidism^10^, atherosclerosis^11^, nonalcoholic fatty liver disease^12^, and preeclampsia^13^, has been investigated. Especially, serum irisin level was tightly related with metabolic diseases and activation of *FNDC5* showed beneficial clinical effects in animal and human^8^,^9^,^14^,^15^,^16^,^17^. In the animal study, *FNDC5* knockout mice showed severe hepatic steatosis with impaired autophagy and fatty acid oxidation. In contrast, *FNDC5* overexpression prevented hyperlipidemia, hepatic lipid accumulation and autophagy impairment in the high fat dieted mouse^16^. In the human study, the patients with type 2 diabetes (T2D) had decreased serum irisin level and T2D drug metformin or glucagon-like peptide-1(GLP-1) treatments increased serum irisin level in the T2D patients^8^,^9^.

Although accumulated evidences suggest that *FNDC5* and irisin play important roles in the regulation of energy metabolism in multiple tissues, the detailed mechanisms for the regulation of expression of these factors remain unknown. Therefore, we investigated the genetic and epigenetic regulation mechanisms of this hormone by using several human tissue samples and hepatocellular carcinoma cell lines that differentially express *FNDC5* variants genes.

## Results

### Differential expression of *FNDC5* in human tissues and cell lines

**Figure 1:**
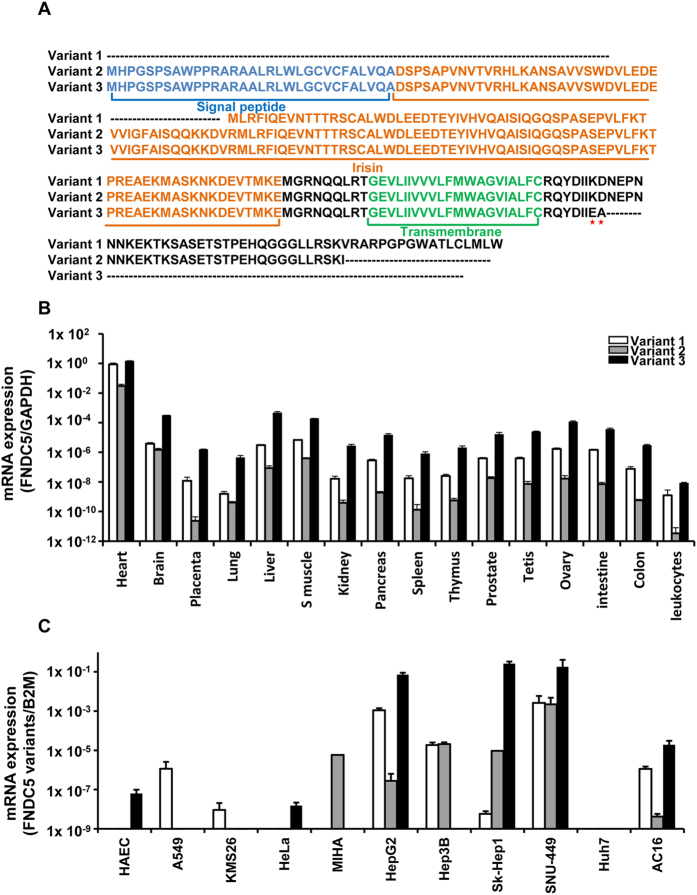
Schematic sequence of human *FNDC5* variants and mRNA expression of *FNDC5* variants in human tissues and cell lines. (**A**) Multiple amino acid sequence alignment of the variants of the human *FNDC5* gene. Variant 1 contains no signal peptide and is cleaved to the irisin sequence. Variants 2 and 3 have a different start codon (ATA) than variant 1, and the C-terminal amino acids are KD → EA in variant 3 (blue = signal peptide, orange = irisin, green = transmembrane sequence). (**B**) The mRNA levels of the three *FNDC5* variants in human tissues are shown. (**C**) The mRNA levels of the three *FNDC5* variants are shown for human cell lines. The expression level of each sample was measured by quantitative real-time RT-PCR using GAPDH or B2M for normalization. HAEC; human aortic endothelial cell, A549; adenocarcinomic human alveolar basal epithelial cells, KMS26; Plasma cell myeloma cell, HeLa; cervical adenocarcinoma cell line, MIHA; nontumorigenic immortalized human hepatocyte cell line, HepG2, Hep3B, Sk-Hep1, SNU449, and Huh7: human hepatoma cell lines, AC16: human cardiomyocyte cell line.

In humans, the *FNDC5* gene has three variants that are distinguished by the signal peptide and C-terminal amino acids (Table 1). Multiple protein sequence alignments of human *FNDC5* gene (Fig. 1A) were used to design unique primers to distinguish the variants in real-time PCR analysis. To determine the tissue specific expression patterns of *FNDC5* variant genes, we performed auantitative real-time PCR of 16 human tissues and 11 human normal or cancer cell lines such as HAEC (human aortic endothelial cell), A549 (adenocarcinomic human alveolar basal epithelial cells), KMS26 (plasma cell myeloma cell), HeLa (cervical adenocarcinoma cell line), MIHA (nontumorigenic immortalized human hepatocyte cell line) HepG2, Hep3B, Sk-Hep1, SNU449, and Huh7(human hepatoma cell lines) and AC16 (human cardiomyocyte cell line).

The real-time PCR results showed different mRNA expression levels for the *FNDC5* gene in several tissues and cell lines (Fig. 1B and C). The highest expression of *FNDC5* mRNA was found in the heart, with higher expression in the brain, liver, skeletal muscle, and ovary compared to other tissues. Expressions of *FNDC5* variants were diverse in different types of normal and cancer cell lines. The human adult cardiomyocyte AC16 and the human hepatocellular carcinoma cell lines including HepG2, Sk-Hep1 and SNU449 showed high mRNA expression level of *FNDC5* variants which were observed in normal heart and liver tissues, while Huh7 cells exhibited extremely low levels of the three *FNDC5* variants. We tested MIHA, an immortalized cell line established from human hepatocytes, as a model of non-tumorigenic normal human hepatocytes. However, the gene expression pattern of *FNDC5* in the cell line was quite different from liver tissues. Based on this screening result, we selected hepatocellular carcinoma cell lines in the proceeding studies to discriminate the transcriptional regulation mechanism of *FNDC5*.

### Modification of the CpG island region within the *FNDC5* promoter correlates with regulation of *FNDC5* expression

**Figure 2:**
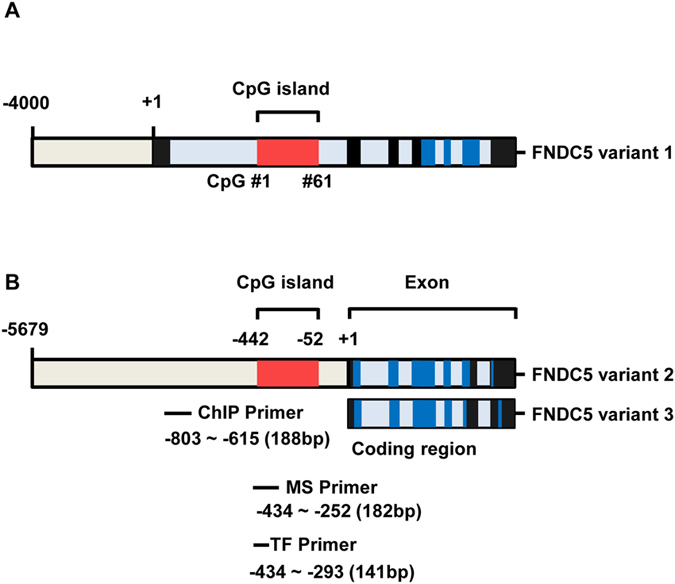
Structure of human FNDC5 variants promoter. (**A** and **B**) The human *FNDC5* variants have different promoter regions. The CpG island in the FNDC5 gene promoter was predicted by “Methprimer” (http://www.urogene.org/cgi-bin/methprimer/Methprimer.cgi). The CpG island is located at −52 to −442 bp in the *FNDC5* promoter, and this region has 61 CpG sites. The CpG island is close to the variant 2 and 3 transcription start sites. Red = CpG island, blue = translational region, black = untranslational region, ChIP primer = chromatin immunoprecipitation primer, MS primer = methylation-specific PCR primer set, TF primer = transcription factor-binding region-specific primer set.

**Figure 3:**
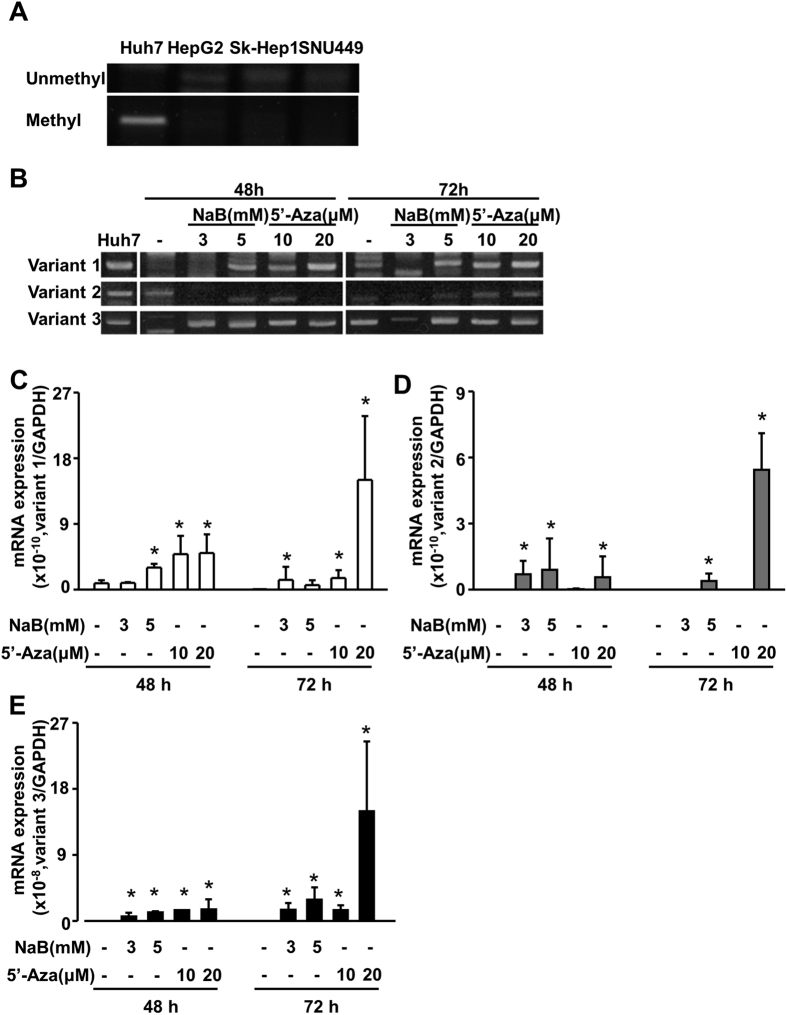
Sodium butyrate (NaB) and 5-azacytidine (5-Aza) increase mRNA expression of *FNDC5* in human hepatocellular carcinoma cell lines. (**A**) Methylation in the CpG island region was measured by methylation-specific PCR. (**B–E**) Huh7 cells were treated with NaB or 5-, and mRNA expression levels of the three *FNDC5* variants were measured by RT-PCR and quantitative real-time PCR.

**Figure 4:**
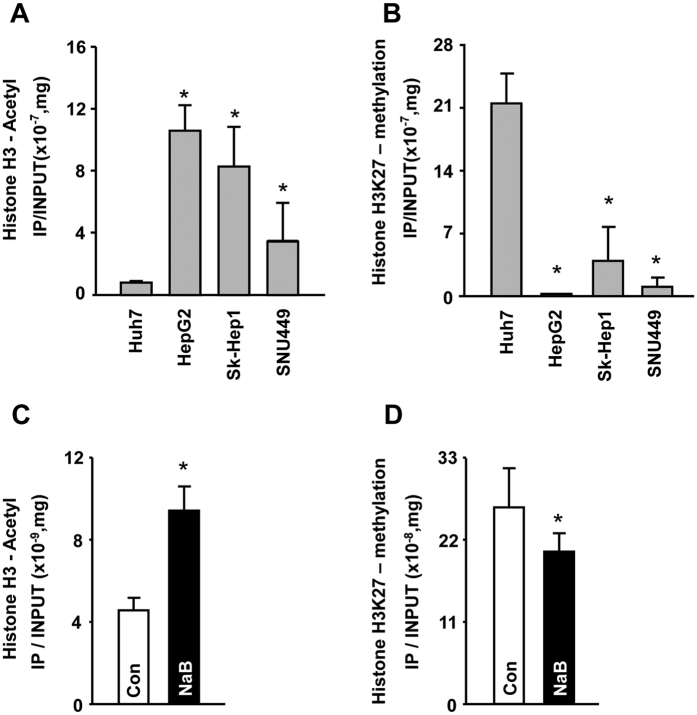
Histone H3 modification at the CpG island in the *FNDC5* promoter is related to the transcriptional regulation of the *FNDC5* gene. (**A** and **B**) ChIP was performed in four cell lines (Huh7, HepG2, Sk-Hep1, and SNU449) using histone H3 acetylation and K27 di-methylation antibodies. (**C** and **D**) ChIP analysis of the CpG island was performed as for (**A**) and (**B**) following treatment of cells with/without 3 mM NaB for 72 h, and expression was measured by quantitative real-time PCR using ChIP primers.

To further elucidate the epigenetic regulation mechanisms involved in *FNDC5* gene expression, we predicted the presence of CpG islands using Meth primer site (http://www.urogene.org/cgi-bin/methprimer/methprimer.cgi). According to this prediction program, a single CpG island region is located at −52~−442 bp of the *FNDC5* variant 2/3 promoter. We designed primers for methylation-specific PCR and chromatin-immunoprecipitation (ChIP) analysis (Fig. 2) to examine the epigenetic modification at the CpG island in four cell lines (Huh7, HepG2, Sk-Hep1, and SNU449). Huh7 cells were highly methylated compared to the other cell lines (Fig. 3A). Next, we determined the mRNA expression levels of the three variants of *FNDC5* following treatment of cells with the histone deacetylase (HDAC) inhibitor sodium butyrate (NaB) or the DNA demethylation agent 5-azacytidine (5-Aza). In Huh7 cells, the mRNA expression of all variants was increased by NaB or 5-Aza treatment (Fig. 3B–E). Based on our previous results, we investigated histone H3 modification using a ChIP assay with H3Ac and H3K27me2 antibodies. Levels of histone H3 acetylation were significantly suppressed in Huh7 cells in comparison to levels in other cell types (Fig. 4A). In contrast to the acetylation levels, Huh7 cells showed significantly greater histone H3 K27 di-methylation levels than the other cell types (Fig. 4B). These data indicate that the epigenetic regulation at histone H3 in the CpG island regulates the mRNA expression of *FNDC5*. Thus, for confirmation, we treated Huh7 cells with 3 mN NaB for 72 h and then performed a ChIP assay with histone H3Ac and H3K27me2 antibodies. NaB-treated cells exhibited higher levels of histone H3 acetylation and lower H3 K27 methylation levels (Fig. 4C and D) in contrast with the first set of ChIP results. These findings confirm that histone H3 modification at the CpG island contribute to the transcriptional regulation of *FNDC5*.

### The core promoter of the *FNDC5* gene spans the region from −1 bp to −1 kb

**Figure 5:**
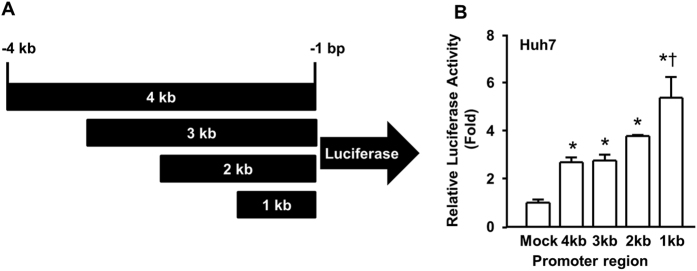
Analysis of *FNDC5* gene expression using an *FNDC5* promoter-driven luciferase reporter plasmid. (**A**) Depiction of the *FNDC5* gene promoter deletion mutants that were used for gene expression analysis. (**B**) Luciferase activity assay with *FNDC5* promoter constructs was performed in Huh7 cells.

To further probe the genetic regulation of *FNDC5* gene expression, we generated *FNDC5* deletion mutants in a luciferase vector (Fig. 5A) for luciferase activity assays in Huh7 cells. Interestingly, the promoter region from −1 bp to −1 kb exhibited high activity compared to that of the other deletion constructs (Fig. 5B). According to these data, the region between −1 bp and −1 kb in the promoter serves as the core regulatory region.

### Glucocorticoid receptor (GR) regulates *FNDC5* gene expression

**Figure 6:**
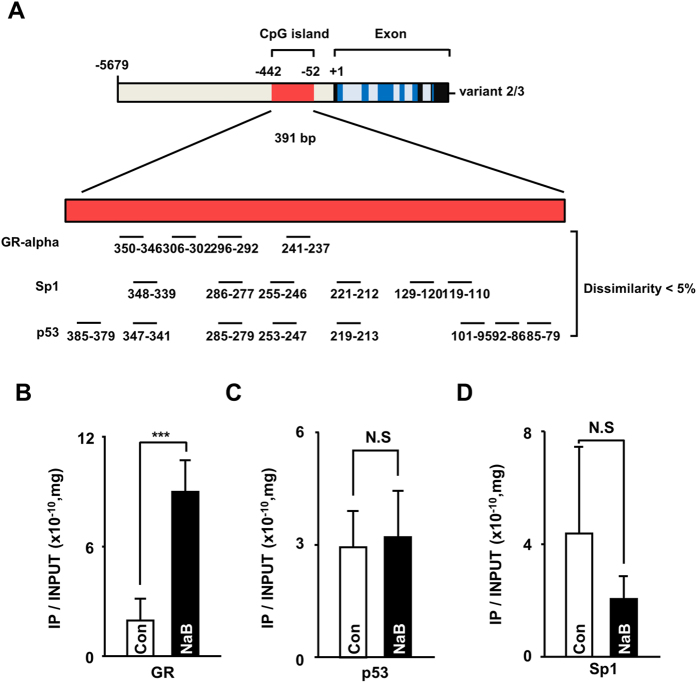
GR is a regulator of *FNDC5* gene expression. (**A**) Schematic of the predicted transcription factor binding sites in the *FNDC5* gene promoter. Prediction of these binding sites was performed using PROMO (http://alggen.lsi.upc.es/cgi-bin/promo_v3/promo/promoinit.cgi?dirDB=TF_8.3) and TF search (http://www.cbrc.jp/research/db/TFSEARCH.html). Each transcription factor has <5% dissimilarity. (**B–D**) ChIP assay in NaB-treated and untreated Huh7 cells with GR-α, p53, and SP1 antibodies. N.S; none significant, ***p < 0.001.

**Figure 7:**
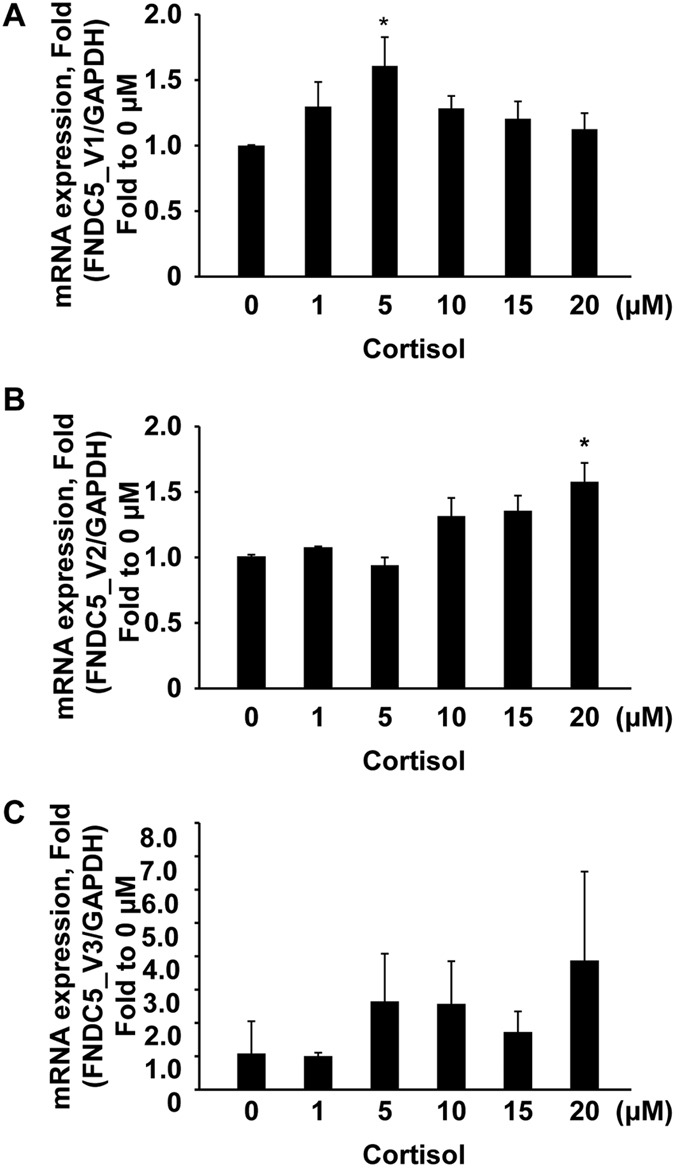
Cortisol increased mRNA expression of *FNDC5* variants 1 and 2 in Huh7 cells. Different doses of cortisol (1–20 μM) treatment significantly increased mRNA expression of *FNDC5* variant 1 (**A**) and variant 2 (**B**) after 24 h. (**C**) Cortisol treatment did not significantly alter *FNDC5* variant 3 expression at any dose.*p < 0.05.

To examine the regulation mechanism associated with the CpG island within the core promoter region (1 bp - 1 kb), we screened transcription factors that were predicted to bind the CpG island in the *FNDC5* gene promoter with the PROMO and transcription factor binding site (TFBS) search analysis tool^18^. Of the identified potential factors, we selected three transcription factors: GR-alpha, Sp1, and p53. The binding site for these transcription factors was abundant and yielded high search accuracies in the CpG island (Fig. 6A). Next, we performed ChIP analysis with these transcription factors in Huh7 cells in the presence or absence of NaB. Interestingly, the GR-alpha binding sites in the CpG island were significantly increased by NaB treatment; however, neither the p53 nor Sp1 binding sites responded to NaB stimulation (Fig. 6B–D). For confirmation, we examined mRNA expression levels following treatment of cells with different doses of cortisol to activate the GR signaling pathway. Cortisol treatment induced increased mRNA expression of *FNDC5* variant 1 and 2 in Huh7 cells (Fig. 7). The effective dose of cortisol required to enhance transcription of *FNDC5* differed between variant 1 and variant 2 and was 5 μM and 20 μM, respectively (Fig. 6A,B). The effect of cortisol on *FNDC5* variant 3, however, was not significant for any treatment dose (Fig. 6C). Thus, the GR-alpha appears to play a role in regulation of *FNDC5* variant expression.

### *FNDC5* expression is regulated similarly in mice

**Figure 8:**
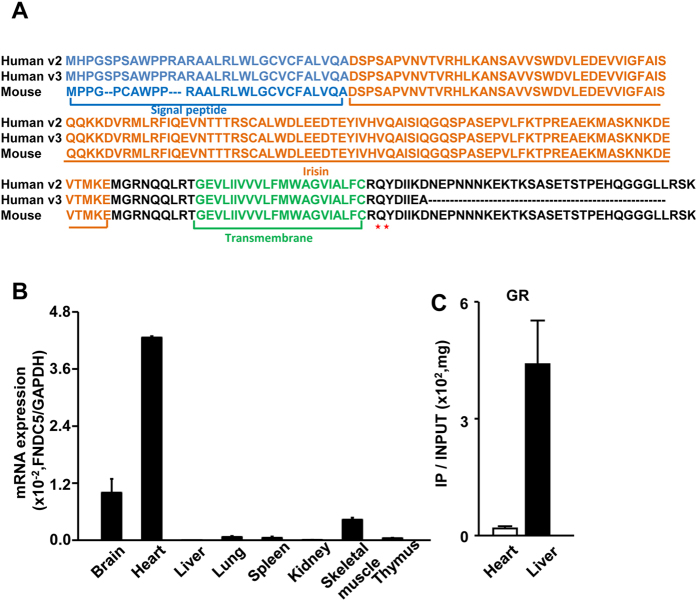
Expression level and regulation of the mouse *FNDC5* gene. (**A**) Amino acid sequence of mouse *FNDC5* was compared with human variants 2 and 3. (**B**) The mRNA expression level of *FNDC5* in mouse tissues. (**C**) ChIP of mouse heart and liver tissue with GR antibody.

To explore the similarities in *FNDC5* regulation between human cell lines and rodents, we first compared the amino acid sequences of *FNDC5* in humans and rodents (Fig. 8A). In mice, no variants were observed, and the sequence showed remarkable similarity with human variant 2. Tissue-dependent mRNA levels of *FNDC5* in mice were investigated using qRT-PCR (Fig. 8B). The observed pattern of mRNA expression in mice is similar to that observed for human variant 2. A mouse tissue ChIP assay was performed to test whether GR also binds *FNDC5* genes in murine heart and liver tissue (C57BL/6, 8w, male, n = 3). ChIP with an anti-GR antibody revealed that GR binds *FNDC5* in liver more strongly than that from heart (Fig. 8C). These findings in mice reflect our findings in human cell lines.

## Discussion

Irisin is generated by cleavage of *FNDC5* and has recently become the focus of much research. Irisin is a myokine that is secreted by skeletal muscle and may be involved in energy and metabolic homeostasis especially in diabetes and obesity^1^,^2^,^3^,^5^,^6^,^7^. Numerous reports have suggested that irisin exerts beneficial effects on metabolic disease and have uncovered the mechanism of the involved pathway; however, these results remain controversial and have not definitely demonstrated the regulation mechanisms^19^. In this study, we investigated the genetic and epigenetic regulation of *FNDC5* gene expression. As an essential finding of the present study, we found that *FNDC5* has 3 different variants, and these variants showed various expression patterns in several types of tissues and cell lines. Remarkably, high expression of *FNDC5* was detected in heart and skeletal muscle tissues. Clearly, the high expression of *FNDC5* in skeletal muscle is well matched with previous reports that *FNDC5* is highly expressed in skeletal muscles and in response to exercise^2^,^6^. Although the latest study showed that *FNDC5* level in rat heart is comparable with skeletal muscle^20^, the high expression of FNDC5 gene in the human heart tissues was not previously reported, with the exception in ‘The Human Protein Atlas (http://www.proteinatlas.org)’. Thus, our result provided valuable information regarding the expression pattern of *FNDC5* variant genes in the human heart. Aside from heart and skeletal muscles, the expression of *FNDC5* was also high in the brain, liver, and ovary. Coincidentally, recent studies have suggested that *FNDC5* is an important mediator for the beneficial effect of exercise on the brain via regulation of brain-derived neurotrophic factor^21^,^22^ and is also a mediator of hepatic glucose and lipid metabolism^23^. The gene expression of *FNDC5* in human ovary has not been reported yet. It only had been reported that the serum irisin level of polycystic ovary syndrome (PCOS) patients were higher compared to the normal control group, however it had been uncertain whether increased irisin level is only a molecular marker for the metabolic syndrome associated with PCOS or whether irisin directly plays a role in ovarian abnormality^24^. The high expression of *FNDC5* gene in our result might give a clue regarding the function of *FNDC5* in reproductive organs. In the energy metabolism aspect, *FNDC5* mRNA expression levels were higher in high energy-dependent tissues (Fig. 1). This distribution of *FNDC5* mRNA expression in energy-dependent tissues suggests that not only the cleaved irisin protein but also the *FNDC5* protein is involved in energy metabolism in humans.

The expression patterns of *FNDC5* variants were diverse in the normal and cancer cell lines. Similar with human tissues, cell lines originating from the heart and liver showed higher expression of *FNDC5* than others. However, liver cell lines (HepG2, Hep3B, Sk-Hep1 and SNU449) expressed much higher *FNDC5* gene expression than heart cell lines (AC16). We also found that the *FNDC5* expressions were different among tissues and matched non-tumor cell lines of heart (AC16) and liver (MIHA), which might be due to transcriptional remodeling during immortalization and adaptation of cells in *in vitro* environments.

Among the tested cell lines, most human hepatocellular carcinoma cells (HepG2, Hep3B, Sk-Hep1, and SNU449) exhibited high mRNA levels of the *FNDC5* variants, although Huh7 cells did not. We therefore determined whether an epigenetic difference in the *FNDC5* gene exists between Huh7 cells and the other cell lines.

CpG islands are short interspersed DNA sequences that play an important role in epigenetic gene transcription initiation. The DNA or histone methylation status in CpG islands is an important regulation factor that determines activation or inactivation of promoters^25^. We investigated the structure of the promoter regions of *FNDC5* variants using the UCSC genome browser, Methprimer, and PROMO sites. We found a single CpG island in the promoter region (−1 bp to −1 kb region), and this CpG island is close to the start codon of variants 2 and 3 (Fig. 2). Analysis of DNA methylation in this CpG island using MS-PCR revealed post-modification of histone H3 or DNA using NaB/5-Aza (Fig. 3). Interestingly, Huh7 DNA was highly methylated compared to the other cell lines, and treatment with the HDAC inhibitor NaB or demethylating agent 5-Aza significantly enhanced the mRNA expression of *FNDC5* in Huh7 cell line. These data indicate that different *FNDC5* mRNA expression levels in human hepatocellular carcinoma cells result from methylation of DNA or histones in the CpG island of the promoter (Fig. 3)^26^. These epigenetic modifications were further tested by comparison of histone H3 acetylation and H3K27 di-methylation levels between Huh7 and the other cells. Both deacetylation and methylation have repressive effects on the transcription of a gene in cancer cells^27^. *FNDC5* expression was clearly lower in Huh7 cells concomitant with a higher level of histone H3K27 methylation and a lower level of H3 acetylation in the CpG island. Moreover, treatment with the HDAC inhibitor NaB significantly increased the acetylation level and decreased the methylation level of the CpG island in the Huh7 cells (Fig. 4).

The role of H3K27me2 has not been as thoroughly studied as that of H3K27me3, which is known to be important for epigenetic regulation. Trimethylation of H3K27 induces inactivation *of* gene expression. In addition, H3K27me1 is associated with activation of promoters^25^,^28^,^29^. A recent study demonstrated that H3K27me2 has a broad effect on promoters and a role in silencing non-cell type-specific enhancers^28^,^29^,^30^. In another study, H3K27me2 was found to be associated with hypo-acetylation^30^ and that acetylation of H3K27 exerts opposite and antagonistic effects to H3K27me3^30^,^31^. These results suggest that *FNDC5* gene transcription is epigenetically regulated by histone H3 acetylation and H3K27 di-methylation.

To identify the major transcription factor promoter binding site, we constructed *FNDC5* promoter deletion mutants in a luciferase vector to detect promoter activity in Huh7 cells (Fig. 5A). Interestingly, the −1 kb region displayed the highest activity compared with the other deletion constructs (Fig. 5B). *S*ubsequently, we searched for key transcription factors that bind to the CpG island within the identified promoter region (−1 kb region). Three transcription factors, GR-alpha, Sp1, and p53, were predicted with a probability >95% to bind to this region and were most frequently observed (Fig. 6A). ChIP assay analysis of these three key transcription factors in the presence or absence of NaB indicated that GR exhibited a higher binding affinity in the NaB-treated group (Fig. 6B–D). In confirmation of this result, we found that cortisol, which can boost GR signaling in Huh7 cells, increased the mRNA expression of *FNDC5* variants 1 and 2. Variant1 mRNA expression was highest at a cortisol dose of 5 μM but decreased at higher doses, while variant 2 mRNA expression gradually increased and then was significantly increased by a cortisol treatment dose of 20 μM. Interestingly, variant 3 did not respond to cortisol treatment at all. These results suggest that different *FNDC5* variants respond differently to cortisol or corticosteroids at the transcriptional level.

Collectively these results indicated that GR-α is a positive regulator of *FNDC5* in human hepatocellular cells and that *FNDC5* transcription may be regulated by cortisol levels. Circulating cortisol levels are increased only via high-intensity exercise training with >60% VO_2_ max levels^32^, and irisin response is also greater during high-intensity exercise than during low-intensity exercise^33^. These studies strongly support the hypothesis that high-intensity exercise enhances circulating cortisol levels and increases GR binding to the *FNDC5* promoter site, resulting in increased transcription of the *FNDC5* gene and consequently increased irisin levels.

Glucocorticoids and the GR are an important genomic regulator that controls the transcription of essential genes involved in development, energy metabolism, immune system, cardiovascular system, and neuronal systems^34^,^35^. Also, GR engages in cross talk with estrogen receptors to reprogram the chromatin configuration^36^. The GR complex binds to target genes and regulates gene expression via glucocorticoid response element (GRE) complex with p300, CBP, PCAF, and SRC that induce histone acetylation^37^,^38^,^39^,^40^. PGC-1a is well known as a master regulator of energy metabolism and a key regulator of irisin and FNDC5^1^,^41^. In addition, PGC-1a is a co-activator of nuclear receptors, including GR, and PGC-1α elevates the binding of GR to its target binding sites^41^,^42^. These results suggest the existence of an inter-relationship among GR, PGC-1α, and FNDC5 regulation. Taken together, these data suggest that GR and cortisol may be potential regulating factors that are involved in exercise-induced PGC-1a and FNDC5/irisin regulation (Fig. 8D).

Although we demonstrated the GR is a possible transcriptional regulator of *FNDC5* in human cell lines and mouse liver tissue, we did not yet test whether exogenous cortisol treatment increases *FNDC5* expression and circulating irisin levels in an animal model. The inter-relationship between cortisol, GR, and *FNDC5*/irisin requires additional investigations in such an *in vivo* setting. Furthermore, the role of cortisol in the exercise-induced irisin elevation should be examined in specific GR-knockout mice in future studies.

## Conclusion

In conclusion, this study revealed a 1-kb core promoter region of the *FNDC5* gene and identified GR as the potential transcription factor of *FNDC5*, which may control the histone acetylation and methylation of the gene. Overall, these finding provide the foundation for understanding the molecular regulatory mechanisms of *FNDC5*/irisin and may lay the groundwork for therapeutic targeting of these factors for treatment of metabolic disease.

## Methods

### Cell culture

Human adult cardiomyocyte AC16 cells, human hepatocellular carcinoma Huh7, HepG2, Sk-Hep1, and SNU449 cells (ATCC), adenocarcinomic human alveolar basal epithelial cells A549, Plasma cell myeloma cell KMS26, cervical adenocarcinoma cell HeLa, nontumorigenic human aortic endothelial cell (HAEC) and nontumorigenic immortalized human hepatocyte cell line (MIHA) were used for this study. All cells were maintained in DMEM/F12 or DMEM supplemented with 10% fetal bovine serum and 1% penicillin/streptomycin. All cells were incubated at 37 °C in an atmosphere of 5% CO_2_ in air and sub-cultured when reaching at least 80% confluences. For experiments, cells were cultured with NaB (Sigma, #B5887) or 5-Aza (Sigma, #A2385) for 24 to 72 h.

### Quantitative Real-time PCR

Total RNA from human cell lines and mouse tissues was extracted using the Trizol^®^ reagent (Ambion, #15596-018) and the RNease Mini kit (Qiagen, #74104), and 1 μg of total RNA of the human first-strand cDNA (Clontech, Human MTC Panel I and II) were transcribed using a first-strand cDNA synthesis kit (Permentas, #K1621). Real time PCR was performed on cDNA with gene-specific primers pairs (Table 2). Reactions were performed in triplicate. The qPCR products were detected with SYBR Green connect Real-Time PCR platform. Results were quantified using the absolute quantitative analysis method via normalization with GAPDH or β-2-mitcroglobulin.

### Luciferase activity assay

*FNDC5* promoter sequences were obtained from UCSC genome browser (https://genome.ucsc.edu/). *FNDC5* promoters were amplified from human genomic DNA (Clontech) with specific primers (Table 3) and inserted into the pGL4.14 vector (Promega) using the KpnI/HindIII enzyme sites and the In-Fusion HD cloning kit (Clontech). The plasmids containing the *FNDC5* promoter and renilla luciferase genes for control were co-transfected using *Lipofectamine*^®^ *LTX* with Plus™ Reagent (Life Technologies). Luciferase activity was measured with a dual luciferase assay kit (Promega). Luciferase activity was normalized to the renilla luciferase activity.

### Methylation-specific PCR

The genomic DNA from cells was extracted using a genomic DNA extraction kit (DNeasy Blood & Tissue kit, Qiagen). As much as 3 μg of gDNA was used for CT conversion with bisulfate treatment (DNA Methylation-Gold kit, Zymo Research) and then amplified with non-methylation- and methylation-specific primers. *FNDC5* nonmethyled primer set (forward 5′–TGGTAGTTAAGTTAGGGTTGTGTATG-3′, reverse 5′–AATAAAAACCAAAAAACACC-3′) and methylation primer set (forward 5′–CGGTAGTTAAGTTAGGGTTGTGTAC-3′, reverse 5′-AAATAAAAACCGAAAAACGC-3′).

### ChIP analysis

The antibodies utilized in ChIP were HitoneH3Ac (Millipore, 06-599), H3K27me2 (Abcam, ab24684), GR (CST, 3660), Sp1 (Santa Cruz, SC-420), and p53 (Santa Cruz, SC-126). ChIP was carried out using a ChIP kit (Active Motif) according to the manufacturer’s specifications, and then the precipitated material was used in quantitative analysis. ChIP was performed with the following CpG island-specific primer pairs: ChIP primer set for human cells (forward 5′-GCAAAGAAAGCTCAGCATAGTATC-3′, reverse 5′-GAGCAGAGAGTGCTAAGTGGAC-3′), ChIP-TF primer set for human cells (forward 5′-CTGTGCACGGGAGAGAGAG-3′, reverse 5′-CTATTAGGTCCTCTGCCGGG-3′), and the ChIP primer set for mouse cells (forward 5′-GATGGGGTACAGATGGGCAT-3′, reverse 5′-GTCCCCGCTCTTCACTCTG -3′).

### Statistical analysis

Data are presented as means ± standard error of the mean (SEM). Differences between the control and treatment groups were evaluated using a one-way analysis of variance (ANOVA), and the control-to-treatment comparison over time or dose was tested using a two-way ANOVA with Origin 8.0 software (Origin Lab). Differences with a *P*-value ≤ 0.05 were considered significant.

## Additional Information

**How to cite this article**: Kyu Kim, H. *et al*. Glucocorticoid receptor positively regulates transcription of *FNDC5* in the liver. *Sci. Rep.* **7**, 43296; doi: 10.1038/srep43296 (2017).

**Publisher's note:** Springer Nature remains neutral with regard to jurisdictional claims in published maps and institutional affiliations.

## Figures and Tables

**Table 1:** Human *FNDC5* variants information.

Variant	NCBI	Amino Acid	NCBI Description
*FNDC5*-1	NP_001165412.1	153	This variant (1) represents the longest transcript and encodes the shortest isoform (1).
*FNDC5*-2	NP_715637.2	212	This variant (2) contains multiple differences in the UTRs and coding region, compared to variant 1. It initiates translation from an alternate “AUA” start codon that is conserved as an “AUG” start codon in orthologs. The encoded isoform (2) is longer and has distinct N- and C-termini, compared to isoform 1.
*FNDC5*-3	NP_001165411.2	181	This variant (3) contains multiple differences in the UTRs and coding region, compared to variant 1. It initiates translation from an alternate “AUA” start codon that is conserved as an “AUG” start codon in orthologs. The encoded isoform (3) is longer and has distinct N- and C-termini, compared to isoform 1.

**Table 2:** Quantitative real-time PCR gene-specific primer sequences.

Gene	Primer sequence
Human *FNDC5* variant 1 3′	TACCAGAGCATGAGGCACAG
Human *FNDC5* variant 2 3′	TTTCATATCTTGCTGCGGAGA
Human *FNDC5* variant 3 3′	ACAGGCAGTCACGCTTCAAT
Human *FNDC5* 5′	CCTCCAAGAACAAAGATGAGG
Human B2M 5′	CTCGCTCCGTGGCCTTAG
Human B2M 3′	CAAATGCGGCATCTTCAA
Rat/Mouse *FNDC5* 3′	GGCTCGTTGTCCTTGATGAT
Rat/Mouse *FNDC5* 5′	GACCTGGAGGAGGACACAGA
Mouse GAPDH 5′	CACCATCTTCCAGGAGCGAG
Mouse GAPDH 3′	CCTTCTCCATGGTGGTGAAGAC

**Table 3:** Primers used for *FNDC5* promoter construction.

Cloning region	Primer sequence
*FNDC5* V1 prom1.5F	CTAACTGGCCGGTACCAAAAGAATGAAACTCCGTCTC
*FNDC5* V1 prom2.5F	CTAACTGGCCGGTACCTCTCAAGTTAATTACCTTTTC
*FNDC5* V1 prom1F	CTAACTGGCCGGTACCGGAAGAATGATTTTTTTTCCA
*FNDC5* V1 prom2F	CTAACTGGCCGGTACCGTGAGCCCTGAGCCCTGGATC
*FNDC5* V1 prom3F	CTAACTGGCCGGTACCACCCAGCCACTCTCCACCACA
*FNDC5* V1 prom4F	CTAACTGGCCGGTACCGCAGCTGAGAATCTGTCTCCA
*FNDC5* V1 promR	CCGGATTGCCAAGCTTCTTTCTCTCCCCTTATTATCT
*FNDC5* prom1DR	CCGGATTGCCAAGCTTTGGAAAAAAAATCATTCTTCC
*FNDC5* prom2DR	CCGGATTGCCAAGCTTGATCCAGGGCTCAGGGCTCAC
*FNDC5* prom3DR	CCGGATTGCCAAGCTTTGTGGTGGAGAGTGGCTGGGT
